# Viscoelastic Polyurethane Foams for Use in Seals of Respiratory Protective Devices

**DOI:** 10.3390/ma14071600

**Published:** 2021-03-25

**Authors:** Małgorzata Okrasa, Milena Leszczyńska, Kamila Sałasińska, Leonard Szczepkowski, Paweł Kozikowski, Katarzyna Majchrzycka, Joanna Ryszkowska

**Affiliations:** 1 Department of Personal Protective Equipment, Central Institute for Labour Protection–National Research Institute, Wierzbowa 48, 90-133 Łódź, Poland; kamaj@ciop.lodz.pl; 2 Faculty of Materials Science and Engineering, Warsaw University of Technology, Wołoska 141, 02-507 Warszawa, Poland; milena.leszczynska.dokt@pw.edu.pl (M.L.); joanna.ryszkowska@pw.edu.pl (J.R.); 3 Department of Chemical, Aerosol and Biological Hazards, Central Institute for Labour Protection–National Research Institute, Czerniakowska 16, 00-701 Warszawa, Poland; kasal@ciop.pl (K.S.); pakoz@ciop.pl (P.K.); 4 FAMPUR Adam Przekurat Company, Gersona 40/30, 83-305 Bydgoszcz, Poland; leonardosz@interia.pl

**Keywords:** viscoelastic polyurethane foams, respiratory protective devices, personalization, leak tightness

## Abstract

A key factor in effective protection against airborne hazards, i.e., biological and nonbiological aerosols, vapors, and gases, is a good face fit of respiratory protective devices (RPDs). Equally important is the comfort of use, which may encourage or discourage users from donning RPDs. The objective of the work was to develop viscoelastic polyurethane foams for use in RPD seals. The obtained foams were characterized using scanning electron microscopy, infrared spectroscopy, thermogravimetry, and differential scanning calorimetry. Measurements also involved gel fraction, apparent density, air permeability, elastic recovery time, compression set, rebound resilience, and sweat uptake. The results were discussed in the context of modifications to the foam formulation: the isocyanate index (I_NCO_) in the range of 0.6–0.9 and the blowing agent content in the range of 1.2–3.0 php. FTIR analysis revealed a higher level of urea groups with increasing water content in the formulation. Higher I_NCO_ and water content levels also led to lower onset temperatures of thermal degradation and higher glass-transition temperatures of the soft phase. A decrease in apparent density and an increase in mean pore sizes of the foams with increasing I_NCO_ and water content levels was observed. Functional parameters (air permeability, elastic recovery time, compression set, rebound resilience, and sweat uptake) were also found to be satisfactory at lower I_NCO_ and water content levels.

## 1. Introduction

Respiratory protective devices (RPD) are classified as personal protective equipment, which is used when it is not possible to prevent occupational risk by eliminating hazards at the source or minimizing them by collective protective measures or organizational solutions. The equipment provided to employees must meet the essential health and safety requirements contained in the relevant standards. Nevertheless, even the best RPD does not ensure the required protection if it does not fit. Proper fit is essential to maintain the leak-tightness of the equipment throughout its use, especially in conditions where exposure to respiratory hazards is very high, e.g., during patient care, rescue and firefighting operations, or performing heavy physical work. In such situations, the user is either unaware of the danger or is unable to seal the equipment due to the nature of the professional activities performed.

The problem of ensuring a leak-tight fit and maintaining it throughout RPD duration is being widely discussed in the research literature and industrial practice, especially in regards to negative pressure equipment [1]. During inhalation, negative pressure under the facepiece draws contaminated air into the breathing zone through leaks between the user’s face and the body of the RPD and the inhalation valves (if any). Of special importance is the preservation of airtightness in reusable equipment with very long or indeterminate useful life (e.g., five years in the case of polymeric half- and full-face masks). It has been found that the risk of an inadequate face seal increased with use time from 10% in the first year to 20% in the second year and 25% in the third year [2,3]. This was mainly attributed to body weight gain or loss, reported by as many as 24% of the study participants. Fit problem upon RPD reuse has also been investigated in the context of the COVID-19 pandemic [4]. In particular, it has been found that gender, and age may play a significant role in RPD fitting [5]. For instance, weight gain during pregnancy may affect RPD fit due to the associated changes in the anthropometric features of the face [6].

The problem of the fit of the equipment is directly related to the comfort of its use. In the case of commercially available RPDs, a good seal is achieved by tightening the head harness/head band and/or nose clip. However, the continuous pressure exerted by the facepiece on the skin often leads to indentation, marks, irritation, and, in some cases, chafing [7]. Some users may choose not to use RPDs even if there is an imminent threat to their health or life. Thus, to improve the performance and safety of RPDs, the design process should focus on the human factors [8,9].

Previous results concerning this issue indicate that selecting the right facepiece model, shape, and size to the individual features of the user’s face may considerably enhance the protective performance of RPDs and, by the same token, worker safety. Research efforts in this area currently proceed along two lines: one involving the improvement of respirator fit methods [10,11] and the other one concerning the application of state-of-the-art design and prototyping methods in RPD development [12,13,14,15]. However, those methods are extremely costly, and so their practical applications are limited. An alternative approach is the application of novel material technologies to improve the user comfort of RPDs and facilitate facepiece fitting for individual wearers with different facial dimensions.

An interesting solution in this respect is a filtering facepiece respirator equipped with a profiled elastic foam layer with openings to permit the free flow of air and moisture [16]. During use, the layer remains in direct contact with the user’s face, ensuring a good fit without the need for a nose clip. Another solution involves a thermoplastic copolymer facemask seal with an anatomically defined geometry capable of being custom-fitted to an individual user’s face using a stream of hot air [17]. The seal shape was designed for optimum fit in the face regions which are at greatest risk of inward leakage, i.e., the nose bridge, cheeks, and chin. In the case of reusable half- and full-masks used with filters, the existing solutions aim only at adapting their shape and size to the broadest possible group of users. On the other hand, there are some interesting examples of medical respiratory masks. For instance, Bennet patented a pneumatic cuff with a specially profiled margin which forms an air cushion between the shell of the mask and the user’s face [18]. Another solution involves a respiratory mask with a seal made of a gel substance possessing resilience characteristics corresponding to those of human fat tissue [19].

Of particular note is the idea of designing facepiece seals made of viscoelastic polyurethane foams characterized by relatively slow elastic recovery time, low resilience, and providing favorable comfort properties in the case of prolonged contact with the human body [20]. The only known application of viscoelastic materials in respiratory devices is a mask designed to be used with ventilators in intensive care units. The applied polyurethane foam cuff was reported to ensure a superior seal to the user’s face, especially in regions at high risk of leakages, that is, around the nose and near the eyes, and also in elderly persons and those with facial hair [21].

The application of viscoelastic polyurethane foams in the construction of RPD seals could provide a protective solution that is self-adaptable to the user’s face due to specifically customized recovery time and simultaneously has superior comfort properties as compared to conventional RPDs due to the low resilience. An appropriate selection of components would facilitate the modification of their properties to make those materials more suitable for the intended application. The molecular weight, functionality, and structure of polyols determine the crosslinking density of foams, significantly affecting their mechanical parameters. Moreover, the high viscosity and reactivity of polyols enable the production of foams with high apparent density. In turn, the higher the water content in the formulation, the greater the number of urea bonds, which leads to greater rigidity, compression strength, and lower elasticity. While viscoelastic foams are typically obtained at low isocyanate index values, in the range of 0.60–0.90, very low isocyanate content in the system adversely affects the mechanical properties of the foams [22,23,24,25].

Considering the above, the objective of the paper was to establish the effects of the isocyanate index and the amount of the blowing agent (water) used on the viscoelastic properties of polyurethane foams, which are essential for their application as RPD seals. A few research methods, such as Fourier transform infrared spectroscopy (FTIR), thermogravimetric analysis (TGA), differential scanning calorimetry (DSC), and air permeability, rebound resilience, or sweat uptake, were applied to define their impact on the properties of foams.

## 2. Materials and Methods

### 2.1. Viscoelastic Polyurethane Foams

A series of 12 viscoelastic polyurethane foams were fabricated using the two-component “Classic” system applied in manufacturing practice by the company Fampur (Bydgoszcz, Poland), at different water contents: 3.0, 2.0, and 1.2 php (parts per hundred parts of polyol) and within a broad range of isocyanate index levels (0.6–0.9). Polyol masterbatches (component A) were prepared by placing the substrates: polyols, catalysts, surfactant, and water (proprietary formulation by Fampur company), weighed with an accuracy of 0.1 g (WTC 300, Radwag, Radom, Poland), in a 500 mL disposable polypropylene container. The substrates were mixed to homogeneity using a high-speed stirrer (Siemens 3 MOT 1LA/080, Siemens, Munich, Germany) at 3000 rpm over 120 s. The characteristics of the prepared masterbatches are given in Table 1.

The isocyanate component (B) was polymeric MDI (BorsodChem, Kazincbarcik, Hungary), commercially traded as Ongronat 4040 TR, containing 32.6% of free isocyanate groups at a calculated equivalent value of R_NCO_ = 128.8.

**Figure 1:**
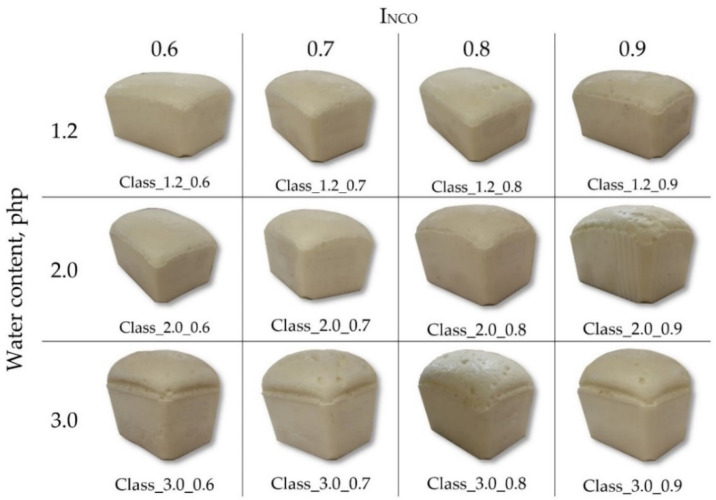
The appearance of the free-rise viscoelastic polyurethane foams.

After adding the isocyanate component to the masterbatch, the system was mixed using a high-speed stirrer at 3000 rpm over 6 s. The mixture was poured into a 1500 mL open polypropylene mold. During the synthesis, the foaming process parameters (start, rise, and gel time) were determined. Subsequently, the foams were cured at 70 °C for 30 min and conditioned at ambient temperature for 22 h before demolding. Before testing, the samples (Figure 1) were conditioned at ambient temperature for at least 72 h.

### 2.2. Synthesis Parameters

An electronic stopwatch was used with an accuracy of 1 s to determine the start time (the time elapsed from the time of combining components A and B to the onset of foam rise), rise time (to maximum foam height), and gel time (to the time when the mixture viscosity is sufficient for pulling a filament from the polymer using a rod).

### 2.3. Gel Fraction

To evaluate the crosslinking level (gel fraction), extraction experiments in *N*,*N*-dimethylformamide (DMF) solvent (Chempur, Piekary Śląskie, Poland) were carried out. Foam samples (four for each type) with initial dry mass (*m_0_*) to approximately 0.1 ÷ 1 g were prepared, weighed, and immersed in DMF for 24 h. Swollen samples were taken out from the solvent. Excess solvent was blotted on cellulose sheets. Afterward, the samples were dried at 60 °C under vacuum until the constant weight was achieved. The weight of the dry samples (*m*) was determined, and the gel fraction was calculated as the ratio between the dry mass of the sample after extraction (*m*) and the dry mass of the sample before extraction *(m_0_)*.

### 2.4. Apparent Density

The apparent density of the foams was determined according to the standard EN ISO 845 [26]. Samples with dimensions 5 cm × 5 cm × 5 cm were measured with an accuracy of 0.1 mm and weighed in the air with an accuracy of 0.001 g using a WPA 180/C/1 scale (Radwag, Radom, Poland).

### 2.5. Scanning Electron Microscopy (SEM)

Samples were observed using a Hitachi SU8010 SEM (Hitachi High-Technology Corporation, Tokyo, Japan) following gold sputtering using a Q150T ES device (Quorum Technologies, Lewes, UK). Imaging was performed with secondary electrons at an acceleration voltage of 5 kV and a working distance of 30 mm. Porosity was assessed in terms of pore size, shape, and spatial distribution based on several images taken at ×30 magnification. SEM images were used to calculate the mean equivalent diameter and aspect ratio of pores (*N* ≥ 500 for each foam variant).

### 2.6. Fourier Transform Infrared Spectroscopy (FTIR)

The absorption spectra of the foams were recorded using a Nicolet 6700 spectrophotometer (Thermo Electron Corporation, Waltham, MA, USA) with an attenuated total reflection (ATR) accessory. Each sample was scanned 64 times in the wavenumber range of 4000–400 cm^−1^. The results were analyzed using Omnic 8.2.0 software (Thermo Fisher Scientific Inc., Waltham, MA, USA).

### 2.7. Differential Scanning Calorimetry (DSC)

DSC was performed to determine the temperature and thermal effects of phase changes using a DSC Q1000 device (TA Instruments, New Castle, DE, USA). Foam samples with a weight of 5.0 ± 0.2 mg were placed in sealed aluminum crucibles, which were initially cooled down to −90 °C, heated to 220 °C at a rate of 10 °C/min (first heating cycle), cooled down again to −90 °C at a rate of 5 °C/min, and finally reheated to 220 °C at a rate of 10 °C/min (second heating cycle). The results were analyzed using Universal Analysis 2000 ver.4.5A software (TA Instruments, New Castle, DE, USA).

### 2.8. Thermogravimetric Analysis (TGA)

Thermogravimetric analysis was done using a TGA Q500 device (TA Instruments, New Castle, DE, USA). Samples with a weight of 10.0 ± 0.5 mg were tested under a nitrogen atmosphere upon heating from ambient temperature to 900 °C at a heating rate of 10 °C/min. The obtained data were analyzed using Universal Analysis 2000 ver.4.7A software (TA Instruments, New Castle, DE, USA).

### 2.9. Air Permeability

Air permeability was determined according to the methodology described in the standard ISO 7131 [27] as the volume of air passed through a 5 cm × 5 cm × 2.5 cm foam sample under a pressure of 125 Pa. Measurements were done in the foam rise and transverse directions for each foam variant (*N* = 4–6).

### 2.10. Elastic Recovery Time, Compression Set, and Rebound Resilience

Elastic recovery time was measured upon the release of a 10 cm × 10 cm × 10 cm sample compressed by 90% for 1 min at ambient temperature; the time was taken with an accuracy of 1 s using an electronic stopwatch [20]. The compression set was determined according to the standard EN ISO 1856 [28]. Compression of 50% and 90% was applied for 22 h at 70 °C to 5 cm × 5 cm × 2.5 cm samples in a direction parallel to the direction of foam growth. Resilience was determined according to the standard EN ISO 8307 [29]. A steel ball with a diameter of 1.6 cm was dropped from a height of 50 cm onto a 10 cm × 10 cm × 10 cm sample cut from the inner part of a foam element. The height of the rebound was measured using slow-motion video analysis.

### 2.11. Sweat Uptake

The uptake of artificial sweat with acidic and alkaline pH was measured for each foam variant. Samples with a weight of 1 g were first dried under vacuum for 12 h at 70 °C and weighed with an accuracy of 0.0001 g to assess dry foam weight (*Wd*) using a Secura 324-1CEU analytical balance (Sartorius, Göttingen, Germany). Each sample was then soaked in a sweat solution for 8 h. Excess fluid was removed from the sample exterior by placing it on fresh filter paper (Thermo Fisher Scientific, Waltham, MA, USA) for 1 min before weighing it to determine the equilibrium swelling weight (*Ws*). The equilibrium weight swelling ratio (*Q*) was calculated as the equilibrium swelling weight divided by the dry foam weight. The sweat solutions were prepared according to ISO 105-E04:2013 [30].

## 3. Results and Discussion

### 3.1. Synthesis Parameters

Irrespective of water content, the start time decreased with increasing I_NCO_. In the case of foams with a water content of 1.2 and 2.0 php, the start time remained in the range of 13–22 s. In turn, foams with a water content of 3 php exhibited a much shorter start time (3–6 s, see Table 2). Furthermore, it was found that the higher the I_NCO_, the longer the rise and gel time (Table 2).

The shortening of the start time is related to the faster reaction rate of the isocyanate with water compared to the rate of reaction of OH groups in secondary and tertiary alcohols (polyols) [31]. Given the intended purpose, it was essential to identify a system with a start time enabling the casting of a mixture of components A and B in the mold and with a short demolding time to optimize the process of seal manufacture on a large scale. From this point of view, it would be beneficial to apply formulations with a water content of 1.2 or 2.0 php and a low isocyanate index; alternatively, the system could be modified to reduce the gel time.

### 3.2. Gel Fraction

Gel fraction analysis indicates that the polymerization reaction of each foam formulation was relatively complete (each had a gel fraction in the range of 82–95%; Table 3), which supports the absence of NCO groups, revealed later on, in ATR-FTIR spectra analysis. Irrespective of water content, an increase in gel fraction correlated with increasing I_NCO_. For foam formulations with lower I_NCO,_ each polyol was less likely to be in proximity of the isocyanate group and form a urethane bond, which resulted in increased chain termination and higher-weight fractions of extractables.

### 3.3. Apparent Density

**Figure 2:**
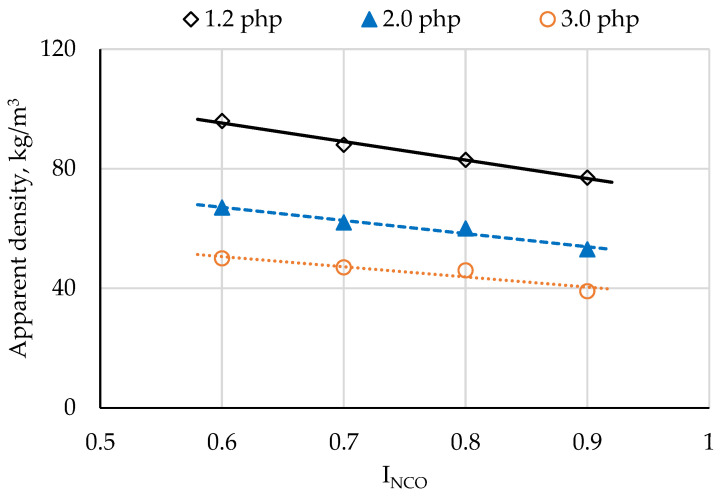
Apparent density depending on isocyanate index and water content.

The apparent density of foams declined with increasing I_NCO_ (from 25% for 1.2 php foam to 28% for 3.0 php foam; Figure 2), as well as with increasing water content in the formulation (on average by up to 89% between 1.2 php and 3.0 php; Figure 2). The decrease in apparent density results from the increase in porosity of the foams with the increase in I_NCO_, which was also demonstrated in their work by Krebs and Hubel [32]. Water is a chemical blowing agent: by increasing its content in the formula of foams, the porosity of the foams increases, which results in a decrease in their apparent density [33].

The facepieces of elastomeric half- and full-face respirators are usually made of silicone, neoprene, ethylene propylene diene monomer (EPDM) rubber or proprietary elastomers with densities ranging from 900 to 2000 kg/m^3^ [34]. The materials produced in this study were characterized by low apparent densities below 100 kg/m^3^. As a result, their application would considerably decrease the overall weight of RPDs, which would be very beneficial both in terms of user comfort and protection. In general, manufacturers aim for more lightweight constructions as any additional weight may impose an ergonomic burden that could translate into cardiac stress, increased energetic cost, musculoskeletal strain, and general discomfort [35,36]. Facepiece weight is also relevant to user safety as the heavier it is, the greater the likelihood of fit issues. In the case of heavy RPDs, wearers often need to manually adjust their fit (e.g., using elastic straps) each time the respirator is donned, which engenders a greater risk of user error [37,38].

### 3.4. Scanning Electron Microscopy (SEM)

**Figure 3:**
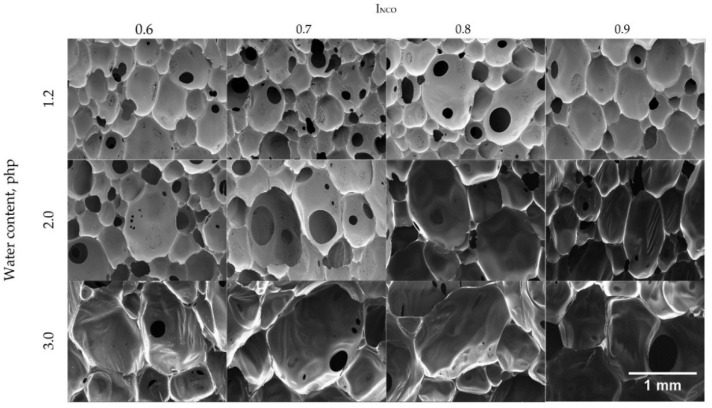
SEM images of foams depending on water content and isocyanate index.

Figure 3 shows the microstructure of foams produced from formulations with different I_NCO_ and blowing agent levels.

SEM images reveal differences in pore size and appearance depending on the isocyanate index and the amount of the blowing agent used. Formulation modifications affected pore size homogeneity (standard deviation from 176 µm for Class_1.2_0.7 to 513 µm for Class_3.0_0.7) as well as pore wall thickness and the number of openings in the pore walls. The homogeneity of structural elements has a direct bearing on the homogeneity of the properties of the end products, which is crucial for the intended application of seals. The higher the water content, the larger the pore size, as can be seen from the mean equivalent diameter (d_2_) given in Table 4. The smallest pore size was found for foams containing 1.2 php of water and the largest for those with the highest water content; this effect was magnified at higher I_NCO_ levels. In the case of the formulation with a water content of 2.0 php, the pore size increased with I_NCO_ in a nearly linear manner. In turn, for foams containing 3.0 php of water, an increase in I_NCO_ in the range of 0.7–0.9 did not have a major effect on the pore size compared to the change from 0.6 to 0.7 (Table 4). An opposite pattern was found for materials with a water content of 1.2 php, which did not exhibit a pronounced increment in pore size until exceeding an I_NCO_ of 0.8. In foams with 1.2 and 2.0 php of water, the pores were oval, while those in foams with 3.0 php of water were polyhedral (Table 4). According to Antunes et al., during foam rise, pores change in shape from spherical to polyhedral when the pore content exceeds approximately 74% [39]. That threshold is probably reached earliest in foams containing 3.0 php of water, and their pore shapes are retained due to the shortest gel time. The most elongated pores were found in Class_2.0_0.7 and Class_2.0_0.9 foams, and the least elongated were in Class_1.2_0.7 and Class_1.2_0.8 foams. In turn, foams with 3 php of water revealed the smallest effect of the isocyanate index on pore shape evolution.

### 3.5. Fourier Transform Infrared Spectroscopy (FTIR)

**Figure 4:**
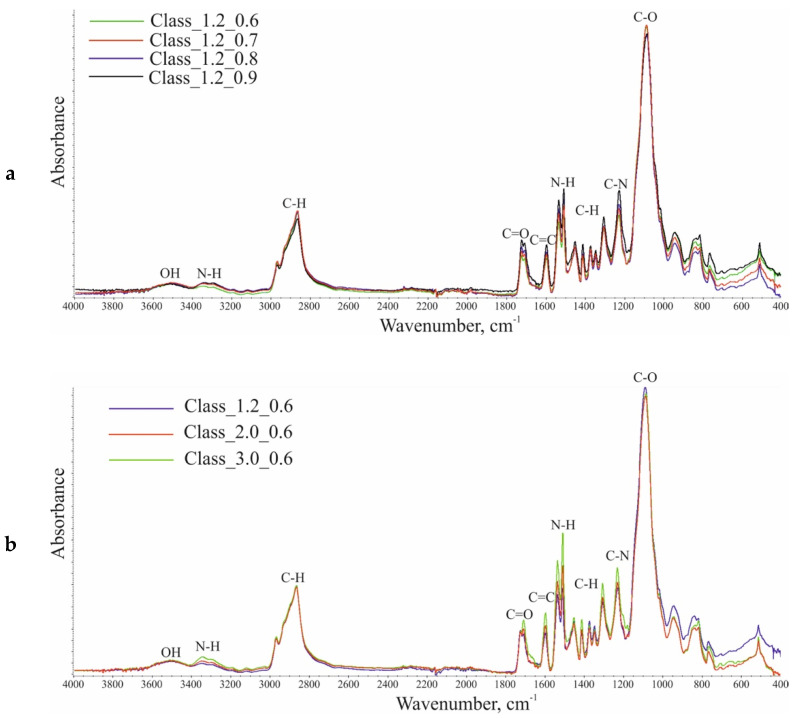
Comparison of ATR-FTIR (attenuated total reflection, Fourier transform infrared spectroscopy) spectra for: (**a**) foams with different I_NCO_ levels at 1.2 php of water and (**b**) foams with different water contents at I_NCO_ = 0.6.

Examples of FTIR spectra for the fabricated foams are given in Figure 4.

FTIR revealed bands characteristic of polyurethanes, which indicate that the synthesis proceeded as planned. The broadband in the wavenumber range of 3600–3400 cm^−1^ arises from the symmetric stretching vibrations of –OH groups in polyols, which are formed at isocyanate index values below 1.0. In 3400–3200 cm^−1^, there is a broad peak resulting from asymmetric and symmetric stretching vibrations of -N-H bonds in urethane groups and urea derivatives. The bands with maxima at 1536 cm^−1^ and 1510–1509 cm^−1^ reflect the deformation vibrations of those bonds. The bands at 2969–2967 cm^−1^ and 2867 cm^−1^ are assigned to asymmetric and symmetric stretching vibrations of CH bonds in CH_3_ and CH_2_ groups. Those groups also give rise to bands at 1453–1452 cm^−1^ (CH_3_) and 1373 cm^−1^ (CH_2_) associated with asymmetric and symmetric deformation vibrations. The multiplet signals at 1725–1724 cm^−1^ and 1711–1708 cm^−1^ reveal the presence of carbonyl bonds (C=O) in the urethane and urea groups. The signals at 1597 cm^−1^ and 767 cm^−1^ indicate the presence of the stretching vibrations of C=C bonds and the out-of-plane vibrations of C-H bonds in aromatic rings. The band at 1230–1228 cm^−1^ is assigned to the stretching vibrations of C-N bonds. The signal with a maximum at 1090–1083 cm^−1^ results from the stretching vibrations of C-O-C groups in the soft segments of polyurethane (macromolecules derived from polyols). The FTIR spectrum did not reveal a signal at a wavenumber of 2270 cm^−1^, which indicates a complete conversion of NCO groups [20,40,41,42].

The increasing level of the water content in the formulation led to a change in the shape of the multiplet signal arising from the vibrations of carbonyl groups, which extended toward lower wavenumbers, which indicates a higher content of urea groups. The results also showed a lower ratio of the intensity of the stretching vibrations of hydroxyl groups compared to that of the asymmetric and symmetric stretching vibrations of -N-H groups.

### 3.6. Differential Scanning Calorimetry (DSC)

**Figure 5:**
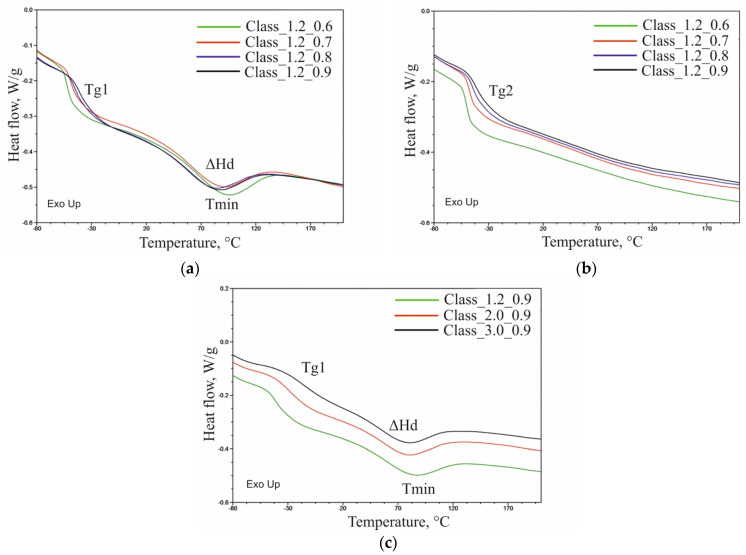
DSC thermograms obtained: (**a**) in the first heating cycle for Class_1.2_0.6–0.9 foams, (**b**) in the second heating cycle for Class_1.2_0.6–0.9 foams, and (**c**) in the first heating cycle for Class_1.2–3.0_0.9 foams.

The DSC curves obtained for foams in the first heating cycle revealed an inflection characteristic of glass-transition temperature (Tg_1_) in the soft polyurethane phase and an endothermic peak attributable to the transformation order–disorder in polyurethane with an extreme temperature T_min_ and a change in enthalpy (ΔHd) [43]. The second heating cycle showed only an inflection typical of glass-transition temperature (Tg_2_). DSC results are given in Table 5 with examples of DSC thermograms presented in Figure 5.

Both in the first and second heating cycles, the glass-transition temperature increased with the isocyanate index and water content. This indicates that both those factors limited the mobility of the segments of the flexible macromolecules that make up the soft phase of the polyurethane. Increasing Tg with increasing I_NCO_ can be the result of an increasing proportion of stiff segments in macromolecules, but it may also be the result of an increase in crosslink density. However, when more water is used in macromolecules, more urea bonds are formed, increasing their stiffness and increasing Tg.

The glass-transition temperature for foams made with 3.0 php of water was higher in the first heating cycle compared to the second cycle. During the synthesis of these foams, the time of individual stages of the process was significantly shorter compared to foams containing 1.2 and 2.0 php of water. In those made with 3.0 php of water, the time for phase separation processes was much shorter. This resulted in a large number of rigid segments remaining dispersed in the soft phase of the foams. The much lower Tg_2_ indicates that further phase separation took place during the annealing, and, consequently, the number of rigid segments dispersed in the soft phase decreased. No differences in Tg_1_ and Tg_2_ were found for foams made with 1.2 and 2.0 php of water. The time of the subsequent stages of the synthesis process of this group of foams was sufficient for phase separation to take place in these materials.

An increase in I_NCO_ led to lower ΔHd for the foam series made with 1.2 and 2.0 php of water, which indicates that for foams prepared using higher INCO smaller number of rigid segments was affected by the order–disorder transformation. This is probably the result of an increase in crosslink density, as more nodes in the network limit macromolecule segments’ mobility. The change in ΔHd is imperceptible in the case of foams made with 3.0 php of water when increasing I_NCO_. In this group of foams, the time for the phase separation process was short, and, consequently, despite the increase in I_NCO_, the number of rigid segments changing their arrangement did not increase.

Analysis results indicate a shift of the endothermic extreme of the peak toward a lower temperature with higher I_NCO_ values and higher water content in the formulation [20,32].

### 3.7. Thermogravimetric Analysis (TGA)

**Figure 6:**
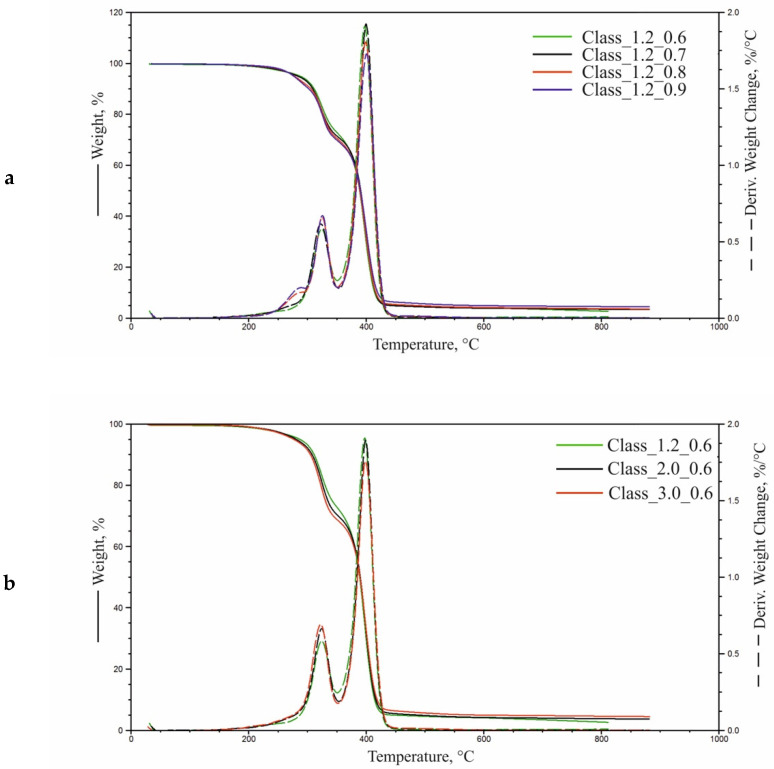
Thermogravimetric analysis (TGA) curves for (**a**) foams with different I_NCO_ levels at a water content of 1.2 php and (**b**) foams with different water contents at I_NCO_ = 0.6.

Figure 6 presents TGA results for foams fabricated at different isocyanate index values and water content levels.

Derivative thermogravimetric (DTG) curves for foams produced at an I_NCO_ of 0.6 and 0.7 reveal two distinct degradation stages, while those with higher I_NCO_ levels show three stages. With increasing I_NCO_, the degree of separation of the hard phase from urethane and urea bonds increases, showing the three stages of degradation. The DTG curves clearly show the degradation stage in the range 240–300 °C. The temperature of 5% weight loss (T_5%_) and residual mass at 600 °C (R_600_) was established from thermogravimetric (TG) curves. In turn, DTG curves were used to determine mass changes in successive degradation stages (Δm, Δm_1_, Δm_2_), maximum degradation rates in stage 2 (V_max1_) and stage 3 (V_max2_), and the temperatures at which those rates were reached (T_max1_ and T_max2_, respectively). The results are given in Table 6.

Thermogravimetric analysis shows that the onset temperature of thermal degradation (T_5%_) decreased with increasing I_NCO_ and water content. This is attributable to greater proportions of hard segments (and especially polyurea), resulting from a higher isocyanate content in the formulation. It should be noted that urea groups exhibit lower thermal stability compared to urethane [33]. Polyurea arises from an isocyanate reacting with water; therefore, its formation is promoted by more water in the reaction mixture.

The thermal degradation of foams with I_NCO_ 0.6 proceeds in two stages with maxima at T_max1_ and T_max2_. The T_max1_ peak represents a multiplet band attributable to the thermal degradation of urea and urethane bonds in hard segments [20,44,45,46], while the band with a peak at T_max2_ arises from the thermal degradation of soft segments [20,44,45,46]. Higher isocyanate index values give rise to a distinct stage of urea bond decomposition at 200–290 °C. It was found that the higher the I_NCO_ and water content, the higher the weight loss (Δm) in that temperature range. An increase in water content in the formulation leads to a slight increment in Δm_1_, which tends to decline with increasing I_NCO_ levels. In this stage, the maximum degradation rate temperature is in the range of 321–325 °C. The higher the water content in the formulation, the higher the maximum degradation rate V_max1_ at T_max1_. Results also indicate a slight increment in that value with increasing I_NCO_. An inverse relationship was found for V_max2_ with T_max2_ with the maximum degradation rate temperature being 397–400 °C. The residual mass of foams upon combustion (R_600_) was 4–6% and slightly increased with water and I_NCO_ levels in the formulation.

### 3.8. Air Permeability

The air permeability of the developed materials is crucial due to their potential application as facepiece seals in RPDs. This parameter should be large enough to ensure user comfort for up to 4 h of skin contact but not too high, lest it should cause the inward leakage of contaminants into the facepiece. Air permeability for the tested samples was below 6 L/min (Table 7), which is a relatively low value compared to foams described in the literature [43,44]. As expected, air permeability increased with water content in the formulation and the associated higher proportion of open pores in the foams. An opposite relationship was found for I_NCO_ due to its negative correlation with the number of pores, as can be seen from SEM images.

### 3.9. Elastic Recovery Time, Compression Set, and Rebound Resilience

Polyurethanes have three-dimensional networks formed as a result of the additive polymerization of their components. Following the compression of such a network, the deformed foam recovers its initial shape due to resilient force. While purely elastic materials do not dissipate energy upon compression, viscoelastic materials do lose energy when a load is applied and then removed (due to plastic deformation). To achieve a slow elastic recovery typical of viscoelastic foams, it is necessary to compensate the elastic force by maximizing three effects that counteract the network effect, that is, the pneumatic, adhesive, and relaxation effects. In elastic polyurethane foams with distinct phases, hard segments do not affect the elasticity of the soft phase. On the other hand, in viscoelastic foams, hard segments are integrated into the structure of the soft phase, and they hinder the mobility of soft segments, causing a delayed recovery to pre-deformation state. Viscoelastic foams are characterized by low rebound resilience, which is less than 20% compared to 25–65% for elastic foams [20,23,32].

**Figure 7:**
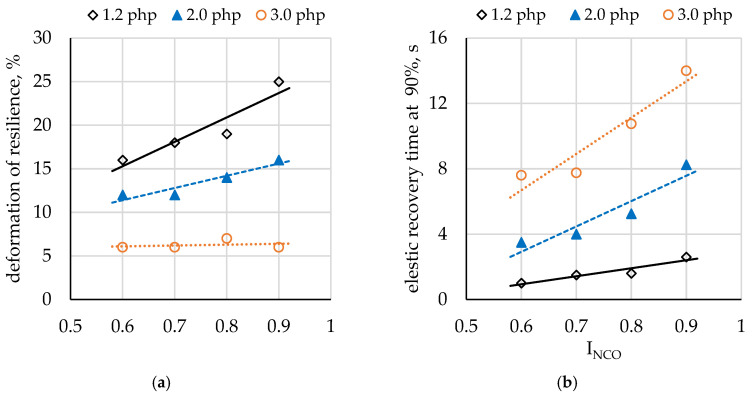
Comparison of (**a**) rebound resilience and (**b**) elastic recovery time for different I_NCO_ and water content levels.

In the materials produced in this study, rebound resilience was found to decrease with increasing water content in the formulation, while an increase in I_NCO_ led to higher resilience values (Figure 7a). Analysis of the results indicates that the developed foams, except for foam Class_1.2_0.9, exhibit resilience values typical of viscoelastic materials. Rebound resilience increased with the isocyanate index for the Class_1.2 and Class_2.0 foam series due to a reduction in the adhesive effect [32]. The absence of that effect for the Class_3.0 series may be attributed to the cellular structure of those foams. The decrease in resilience with increasing water content may be explained by a higher proportion of open pores, thinner pore walls, and larger pore size. These results are consistent with SEM image analysis.

Elastic recovery time at 90% increased both as a result of increasing water content and I_NCO_ in the system (Figure 7b). The unique properties of viscoelastic polyurethane foams arise from incomplete separation of the hard and soft phases, due to which the soft and hard segments remain partially intertwined, causing a delay in recovery to an initial state before deformation. The higher the proportion of hard segments in the foam, the longer the elastic recovery time, which may be associated with a greater number of hard segments embedded in the soft phase. As in the case of rebound resilience, an increase in recovery time with increasing water content may be due to alterations in the cellular structure of the foams. It is thought that recovery time in the range of 2–6 s is most suitable for the practical applications of viscoelastic foams [23]. Thus, the recovery time obtained for the Class_2.0_0.9 foam and the Class_3.0 foam series limits their application potential as RPD seals.

The compression set results given in Table 8 indicate that the foams synthesized with a low isocyanate content in the formulation (I_NCO_ = 0.6) underwent permanent deformation after 90% compression (22 h, 70 °C), which implies that either higher I_NCO_ values should be applied or the formulation should be modified to allow for I_NCO_ = 0.6. The water content of 3 php led to high-compression set values. The other foam compression set either after 50% or 90% compression was suitable for their application as RPD seals.

### 3.10. Sweat Uptake

When RPDs must be worn over long periods, user comfort associated with the materials applied in the equipment becomes a crucial issue. One of the major causes of discomfort connected with wearing RPDs is the accumulation of moisture on the skin under the facepiece [34]. This problem is particularly evident under hard working conditions (e.g., elevated temperature and high humidity), which create a favorable environment for microbial growth [47,48]. To address these issues, it is preferable to use materials with improved sweat sorption properties, capable of removing liquid from the skin surface. The materials developed in this study exhibited very different sweat adsorption properties (Table 9). The equilibrium swelling ratio decreased with increasing I_NCO_ both for acidic and alkaline sweat, which is probably attributable to the decline in the proportion of open pores in the foam structure, hindering liquid uptake. Higher swelling-rate values were found for acidic sweat. Water content did not have a significant difference in sweat uptake.

## 4. Conclusions

A series of 12 viscoelastic polyurethane foams were produced from formulations with different water content levels and a broad range of isocyanate index values. The foams were characterized in terms of their chemical structure as well as physicomechanical, thermal, and functional properties in regard to their intended application as seals in respiratory protective devices. The results provided an orientation for further technological work on the development of facepiece seals. Chemical analysis revealed a higher level of urea groups with increasing water content in the formulation. Higher isocyanate and water content levels also led to lower onset temperatures of thermal degradation and higher glass-transition temperatures of the soft phase. From the standpoint of process parameters, the best formulations were those with a water content of 1.2 or 2.0 php and low isocyanate index values. SEM revealed that higher I_NCO_ and water content values led to the lower apparent density of the foams, and larger mean pore sizes.

These findings were reflected in the results of functional studies, that is, air permeability, elastic recovery time, compression set, rebound resilience, and sweat uptake, which were also found to be satisfactory at lower I_NCO_ and water content levels.

The application of viscoelastic polyurethane foams in the construction of RPD seals enables a universal and customizable solution. The spontaneous and continuous adaptation of the seal shape to the individual features of the face prevents leakage irrespective of the wearer’s awareness or changes in his or her facial features over time. However, it should be noted that the material used in RPDs, intended for use by persons exposed to health and safety hazards, should meet certain requirements concerning mechanical strength and resistance to high temperatures, flames, and chemical substances. Further research aimed at the development of such seals will be conducted taking into account these parameters.

## Figures and Tables

**Table 1:** Characteristics of polyol masterbatches.

Water Content, php	Density (25 °C), g/cm^3^	Viscosity (25 °C), mPas	Hydroxyl Value, mg KOH/g	ROH, g/mol
3.0	1.06	1100	259.8	216.7
2.0	1.05	1200	201.1	279.0
1.2	1.05	1200	154.0	364.2

**Table 2:** Process times for different values of the isocyanate index and water content.

Foam Type	Process Parameters, s		
	Start Time	Rise Time	Gel Time
Class_1.2_0.6	22	240	330
Class_1.2_0.7	18	250	350
Class_1.2_0.8	15	310	400
Class_1.2_0.9	13	330	600
Class_2.0_0.6	20	210	310
Class_2.0_0.7	18	220	320
Class_2.0_0.8	16	230	390
Class_2.0_0.9	14	250	470
Class_3.0_0.6	6	144	260
Class_3.0_0.7	4	160	290
Class_3.0_0.8	3	150	310
Class_3.0_0.9	3	210	440

**Table 3:** Gel fraction of foam samples depending on I_NCO_ and water content.

Foam Type	Gel Fraction, %
Class_1.2_0.6	82.1 ± 2.2
Class_1.2_0.7	88.4 ± 1.7
Class_1.2_0.8	91.4 ± 2.0
Class_1.2_0.9	95.2 ± 3.6
Class_2.0_0.6	85.9 ± 1.4
Class_2.0_0.7	90.7 ± 1.3
Class_2.0_0.8	91.8 ± 3.1
Class_2.0_0.9	94.4 ± 3.4
Class_3.0_0.6	83.4 ± 3.6
Class_3.0_0.7	86.6 ± 2.7
Class_3.0_0.8	89.4 ± 3.2
Class_3.0_0.9	93.5 ± 2.7

**Table 4:** Structural parameters of foams depending on the isocyanate index and water content.

Foam Type	Mean Pore Equivalent Diameter, d₂, μm	Pore Aspect Ratio AR, a.u.
Class_1.2_0.6	302 ± 195	1.53 ± 0.27
Class_1.2_0.7	279 ± 176	1.39 ± 0.25
Class_1.2_0.8	336 ± 268	1.39 ± 0.23
Class_1.2_0.9	473 ± 262	1.55 ± 0.26
Class_2.0_0.6	372 ± 230	1.43 ± 0.25
Class_2.0_0.7	425 ± 255	1.62 ± 0.30
Class_2.0_0.8	468 ± 282	1.53 ± 0.27
Class_2.0_0.9	476 ± 289	1.62 ± 0.30
Class_3.0_0.6	542 ± 412	1.49 ± 0.29
Class_3.0_0.7	693 ± 215	1.47 ± 0.29
Class_3.0_0.8	683 ± 470	1.46 ± 0.25
Class_3.0_0.9	667 ± 485	1.48 ± 0.77

**Table 5:** Differential scanning calorimetry (DSC) results.

Foam Type	Tg_1_, °C	ΔHd, J/g	Tmin, °C	Tg_2_, °C
Class_1.2_0.6	−53	34.8	93	−50
Class_1.2_0.7	−47	29.6	86	−47
Class_1.2_0.8	−45	27.7	80	−45
Class_1.2_0.9	−42	27.0	82	−42
Class_2.0_0.6	−46	31.6	88	−46
Class_2.0_0.7	−42	31.2	90	−42
Class_2.0_0.8	−38	30.2	79	−38
Class_2.0_0.9	−30	25.9	77	−34
Class_3.0_0.6	−39	32.4	86	−44
Class_3.0_0.7	−30	30.3	81	−37
Class_3.0_0.8	−29	31.0	82	−37
Class_3.0_0.9	−16	31.2	76	−29

**Table 6:** Foam parameters determined from thermogravimetric (TG) and derivative thermogravimetric (DTG) curves.

Sample Designation	T_5%_, °C	Δm, %	T_max1_, °C (V_max1_, %/°C)	Δm_1_,%	T_max2_, °C (V_max2_, %/°C)	Δm_2_,%	R_600_, %
Class_1.2_0.6	287	3.2	324 (0.58)	23.3	397 (1.91)	67.7	4.1
Class_1.2_0.7	283	3.4	323 (0.61)	23.4	399 (1.92)	66.0	3.9
Class_1.2_0.8	277	5.5	324 (0.66)	22.2	399(1.81)	64.6	4.2
Class_1.2_0.9	276	7.7	325 (0.67)	21.8	400 (1.72)	63.4	4.9
Class_2.0_0.6	281	3.4	324 (0.67)	25.1	399 (1.89)	64.3	4.9
Class_2.0_0.7	277	4.8	323 (0.70)	24.8	399(1.82)	62.1	5.1
Class_2.0_0.8	275	6.5	324 (0.71)	24.1	399 (1.70)	61.2	5.0
Class_2.0_0.9	271	8.8	325 (0.74)	23.0	399 (1.53)	59.8	5.2
Class_3.0_0.6	277	4.1	322 (0.69)	27.2	399 (1.75)	62.1	5.0
Class_3.0_0.7	274	5.0	321 (0.75)	27.4	400 (1.69)	59.2	5.3
Class_3.0_0.8	272	7.7	322 (0.76)	25.9	398 (1.63)	58.5	5.7
Class_3.0_0.9	268	10.2	322 (0.76)	24.5	397 (1.42)	55.6	5.8

**Table 7:** The air permeability of foam samples depending on I_NCO_ and water content.

Foam Type	Air Permeability at 125 Pa, L/min
Class_1.2_0.6	2.8 ± 0.9
Class_1.2_0.7	0.5 ± 0.1
Class_1.2_0.8	1.2 ± 0.6
Class_1.2_0.9	0.7 ± 0.2
Class_2.0_0.6	4.4 ± 0.8
Class_2.0_0.7	1.4 ± 0.4
Class_2.0_0.8	1.1 ± 0.3
Class_2.0_0.9	0.6 ± 0.1
Class_3.0_0.6	5.5 ± 0.5
Class_3.0_0.7	4.8 ± 1.1
Class_3.0_0.8	5.4 ± 1.2
Class_3.0_0.9	3.7 ± 0.7

**Table 8:** Compression set at 50% and 90% for different I_NCO_ and water content levels.

Foam Type	Compression Set at 50% (22 h, 70 °C), %	Compression Set at 90% (22 h, 70 °C), %
Class_1.2_0.6	4 ± 2	85 ± 1
Class_1.2_0.7	2 ± 1	2 ± 2
Class_1.2_0.8	1 ± 1	1 ± 1
Class_1.2_0.9	1 ± 1	1 ± 1
Class_2.0_0.6	2 ± 1	74 ± 4
Class_2.0_0.7	0 ± 0	1 ± 1
Class_2.0_0.8	1 ± 1	1 ± 1
Class_2.0_0.9	0 ± 0	1 ± 1
Class_3.0_0.6	2 ± 1	85 ± 1
Class_3.0_0.7	1 ± 1	4 ± 2
Class_3.0_0.8	1 ± 1	35 ± 16
Class_3.0_0.9	2 ± 1	13 ± 8

**Table 9:** Comparison of equilibrium swelling ratio for sweat with alkaline and acidic pH depending on I_NCO_ and water content.

Foam Type	Equilibrium Swelling Mass, g_dry_/g_wet_	
	Alkaline Sweat	Acidic Sweat
Class_1.2_0.6	19.7 ± 0.4	19.5 ± 0.6
Class_1.2_0.7	16.0 ± 1.0	18.9 ± 0.9
Class_1.2_0.8	12.5 ± 0.8	13.4 ± 0.7
Class_1.2_0.9	8.1 ± 0.4	10.1 ± 2.7
Class_2.0_0.6	16. 8± 0.5	20.5 ± 0.4
Class_2.0_0.7	12.8 ± 0.4	14.1 ± 0.7
Class_2.0_0.8	11.2 ± 0.5	11.1 ± 0.6
Class_2.0_0.9	9.1 ± 0.5	10.4 ± 1.6
Class_3.0_0.6	18.3 ± 0.8	19.9 ± 0.4
Class_3.0_0.7	14.5 ± 1.7	16.4 ± 1.8
Class_3.0_0.8	15.7 ± 0.6	17.2 ± 2.0
Class_3.0_0.9	11.1 ± 1.0	11.2 ± 0.6

## Data Availability

The data presented in this study are available on request from the corresponding author.
